# Supplementary material for: scPrediXcan integrates advances in deep learning and single-cell data into a powerful cell-type–specific transcriptome-wide association study framework
Source: bioRxiv. 2025 Mar 4:2024.11.11.623049. Originally published 2024 Nov 14. Preprint. [Version 2] doi: 10.1101/2024.11.11.623049 (PMC11601274; doi:10.1101/2024.11.11.623049)
Supplement: Supplement 1 [file media-1.zip › Supplementary_figures/Sup_fig2.pdf]

Supplementary fig. 2: Brief description of the models in the scPrediXcan framework.

Model name	Input	Output	Architecture type
Enformer	200kb DNA sequences	5313*896 epigenomic feature matrix	Transformer-based model
ctPred	5313*1 epigenomic representations	One pseudo-bulk gene expression value	Multilayer perceptron
l-ctPred	Genotype SNP dosages within 1Mb of the gene body	One pseudo-bulk gene expression value	Linear elastic net
