# Supplementary material for: scPrediXcan integrates advances in deep learning and single-cell data into a powerful cell-type–specific transcriptome-wide association study framework
Source: bioRxiv. 2025 Mar 4:2024.11.11.623049. Originally published 2024 Nov 14. Preprint. [Version 2] doi: 10.1101/2024.11.11.623049 (PMC11601274; doi:10.1101/2024.11.11.623049)

Supplementary fig. 6: Quantile-quantile plot of TWAS $-\log_{10}$ (p-value) for only the overlapped genes between models

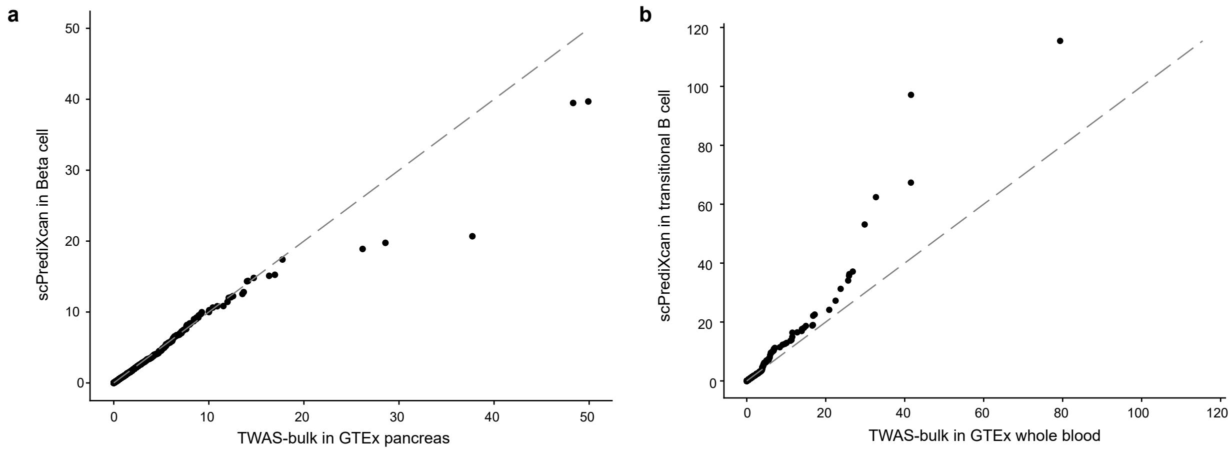

Supplement: Supplement 1 [file media-1.zip › Supplementary_figures/Sup_fig6.pdf]
