# Supplementary figures and images for: scPrediXcan integrates advances in deep learning and single-cell data into a powerful cell-type–specific transcriptome-wide association study framework
Source: bioRxiv. 2025 Mar 4:2024.11.11.623049. Originally published 2024 Nov 14. Preprint. [Version 2] doi: 10.1101/2024.11.11.623049 (PMC11601274; doi:10.1101/2024.11.11.623049)

Supplementary fig. 1: ctPred predicts cell type-specific gene expressions in CD 4+ T cell

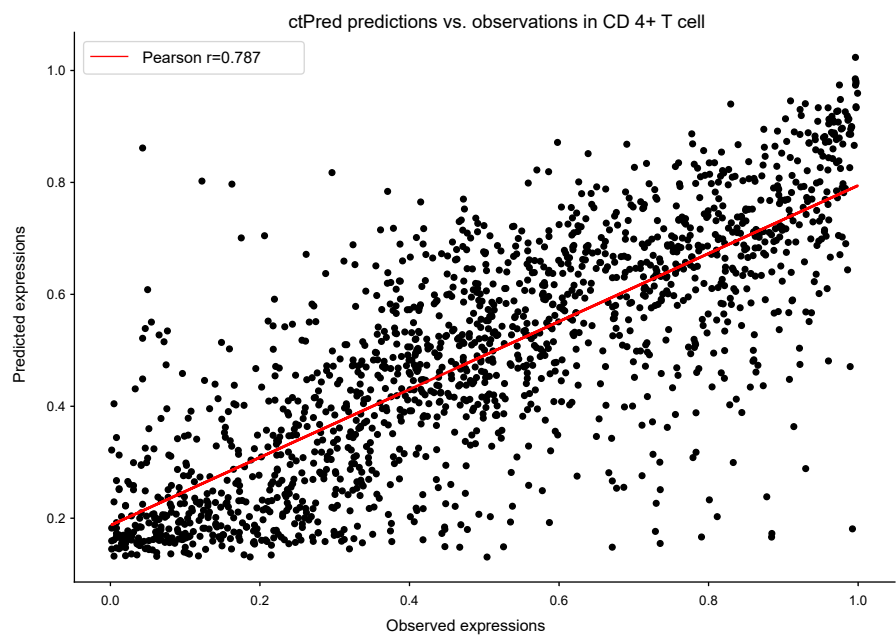

Supplement: Supplement 1 [file media-1.zip › Supplementary_figures/Sup_fig1.pdf]

Supplementary fig. 8: scRNA-seq pseudobulk data processing for ctPred training.

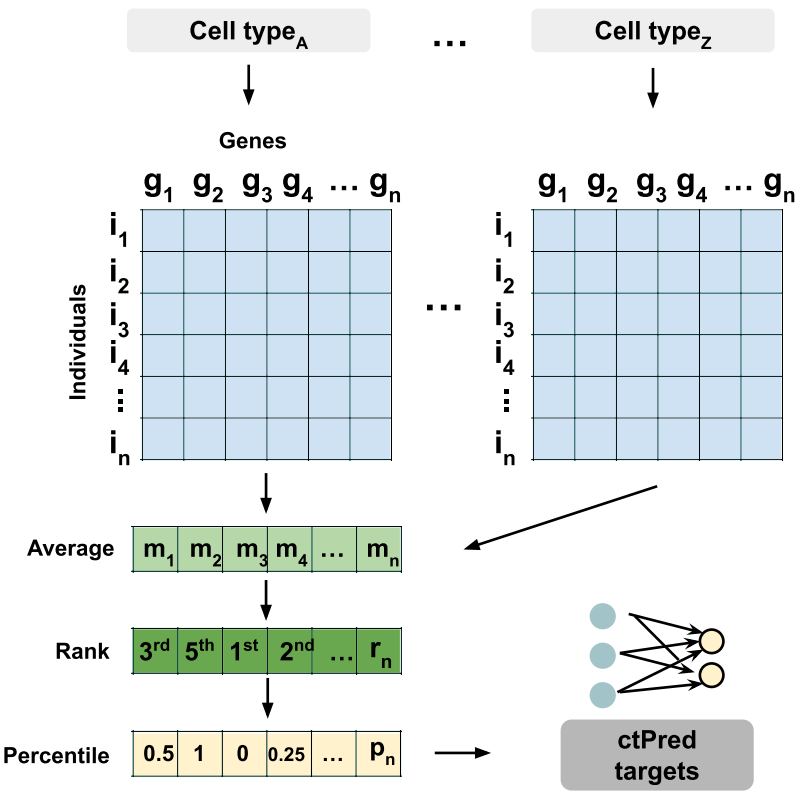

Supplement: Supplement 1 [file media-1.zip › Supplementary_figures/Sup_fig8.pdf]
