# Supplementary material for: scPrediXcan integrates advances in deep learning and single-cell data into a powerful cell-type–specific transcriptome-wide association study framework
Source: bioRxiv. 2025 Mar 4:2024.11.11.623049. Originally published 2024 Nov 14. Preprint. [Version 2] doi: 10.1101/2024.11.11.623049 (PMC11601274; doi:10.1101/2024.11.11.623049)
Supplement: 1 [file NIHPP2024.11.11.623049V2-supplement-1.pdf]

bioRxiv preprint doi: https://doi.org/10.1101/2024.11.11.623049; this version posted March 4, 2025. The copyright holder for this preprint (which was not certified by peer review) is the author/funder, who has granted bioRxiv a license to display the preprint in perpetuity. It is made available under aCC-BY 4.0 International license.

Supplementary information

Supplementary Figures

Supplementary fig. 1: ctPred predicts cell type-specific gene expressions in CD 4+ T cell

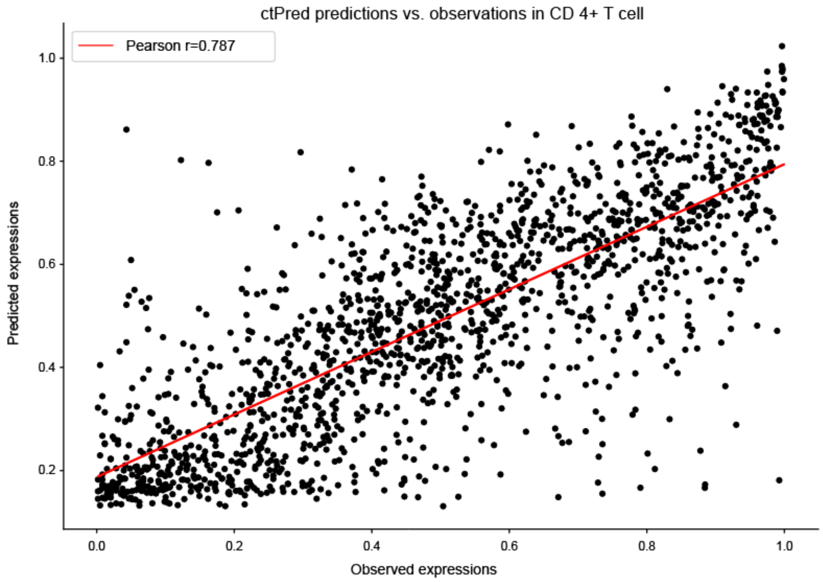

Supplementary fig. 1 Scatter plot of ctPred predictions and observations for gene expressions in CD4+ T cell dataset.

Supplementary fig. 2: Brief description of the models in the scPrediXcan framework.

Model name	Input	Output	Architecture type
Enformer	200kb DNA sequences	5313*896 epigenomic feature matrix	Transformer-based model
ctPred	5313*1 epigenomic representations	One pseudo-bulk gene expression value	Multilayer perceptron
l-ctPred	Genotype SNP dosages within 1Mb of the gene body	One pseudo-bulk gene expression value	Linear elastic net

bioRxiv preprint doi: https://doi.org/10.1101/2024.11.11.623049; this version posted March 4, 2025. The copyright holder for this preprint (which was not certified by peer review) is the author/funder, who has granted bioRxiv a license to display the preprint in perpetuity. It is made available under aCC-BY 4.0 International license.

Supplementary fig. 3: Quantile-quantile plot of ACAT-adjusted TWAS -$\log_{10}$ (p-value) against uniformly distributed p-value for T2D and SLE.

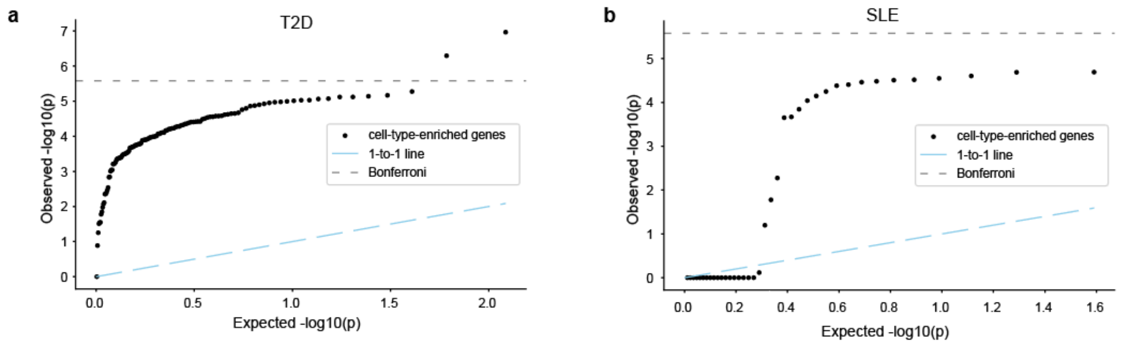

Supplementary fig. 3 a) Quantile-quantile plot of ACAT-aggregated TWAS -log10 (p-value) in all non-significant cell types for genes passing the Bonferroni-corrected threshold in only one islet cell type from T2D dataset for T2D trait. **b)** Quantile-quantile plot of ACAT-aggregated TWAS -log10 (p-value) in all non-significant cell types for genes passing the Bonferroni-corrected threshold in only one immune cell type from OneK1K dataset for SLE trait.

Supplementary fig. 4: Calculating the number of true positive genes with p-values deviating from a uniform distribution.

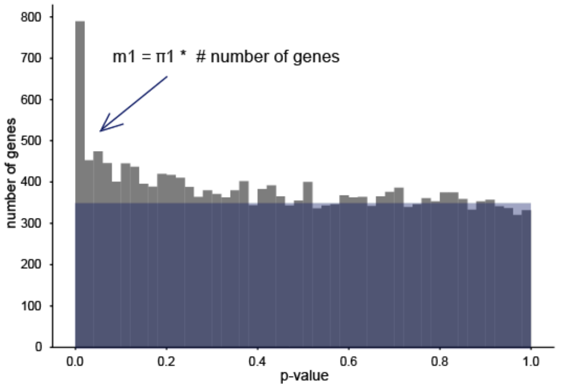

Supplementary fig. 4 The m1 calculation is based on the histogram of p-values of Pearson correlations between predicted gene expressions and observed gene expressions. The blue rectangular region shows the genes with p-values following a uniform distribution, and the proportion of those genes is denoted as pi0. The pi1 is 1-pi0, and the number of true positive genes is calculated by pi1 * total number of genes.

bioRxiv preprint doi: https://doi.org/10.1101/2024.11.11.623049; this version posted March 4, 2025. The copyright holder for this preprint (which was not certified by peer review) is the author/funder, who has granted bioRxiv a license to display the preprint in perpetuity. It is made available under aCC-BY 4.0 International license.

Supplementary fig. 5: Distribution of Spearman correlations between ctPred predictions and l-ctPred predictions of all genes in representing cell types

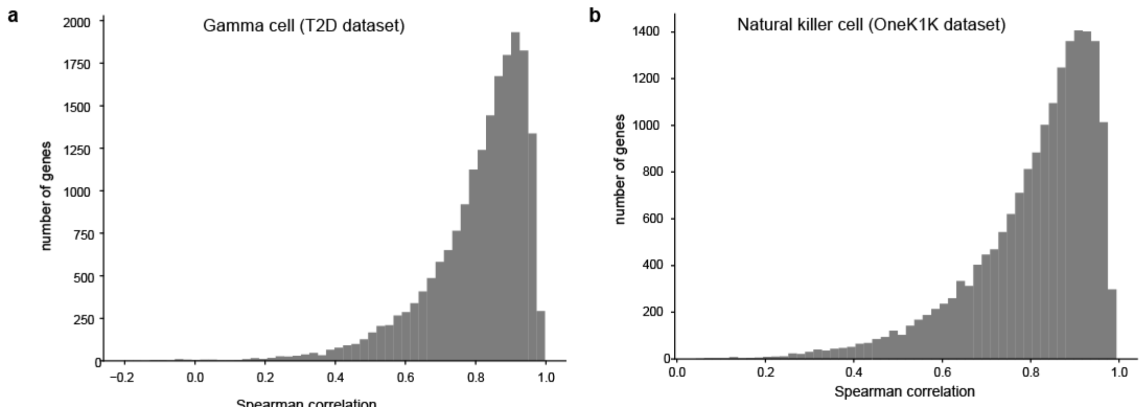

Supplementary fig. 5 a) Histogram of Spearman correlations between ctPred-predicted gene expressions and l-ctPred-predicted gene expressions in gamma cell as the representing cell type from T2D dataset. Other cell types have similar correlation distributions. **b)** Histogram of Spearman correlations between ctPred-predicted gene expressions and l-ctPred-predicted gene expressions in natural killer cells as the representing cell type from OneK1K dataset. Other cell types have similar correlation distributions.

Supplementary fig. 6: Quantile-quantile plot of TWAS -$\log_{10}$ (p-value) for only the overlapped genes between models

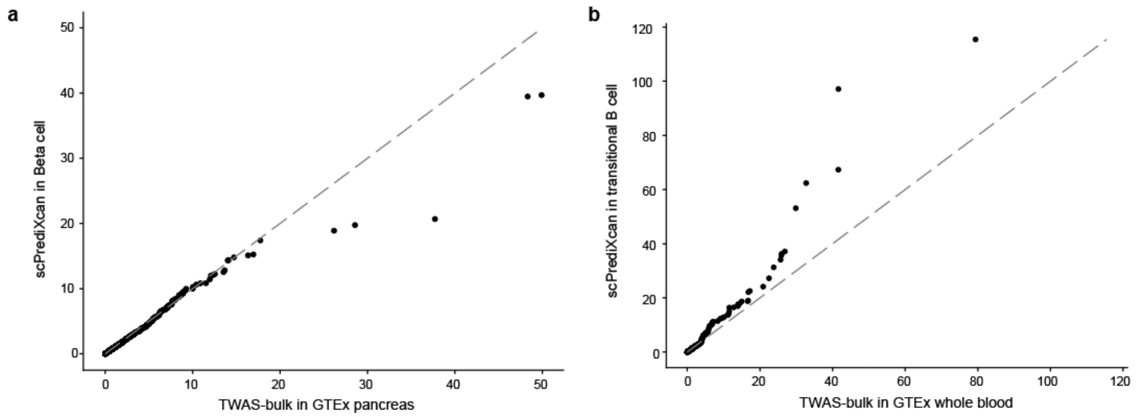

Supplementary fig. 6 a) Quantile-quantile plot of T2D TWAS -log10(p) of overlapped genes in scPrediXcan in Beta cell and TWAS-bulk in GTEx pancreas. This set of genes will likely favor the TWAS-bulk method since only models that performed well enough in this approach end up included here. A more fair comparison is shown in Figure 5b where union of genes tested by scPrediXCan and TWAS-bulk in GTEx pancreas are shown, imputing the p-values of genes missed by TWAS-bulk with uniformly distributed p-values. **b)** Quantile-quantile plot of SLE

bioRxiv preprint doi: https://doi.org/10.1101/2024.11.11.623049; this version posted March 4, 2025. The copyright holder for this preprint (which was not certified by peer review) is the author/funder, who has granted bioRxiv a license to display the preprint in perpetuity. It is made available under aCC-BY 4.0 International license.

TWAS -log10(p) of overlapped genes in scPrediXcan in transitional B cell and TWAS-bulk in GTEx blood.

Supplementary fig. 7: ctPred prediction performance metrics vs. cell numbers and read counts of different cell types across datasets.

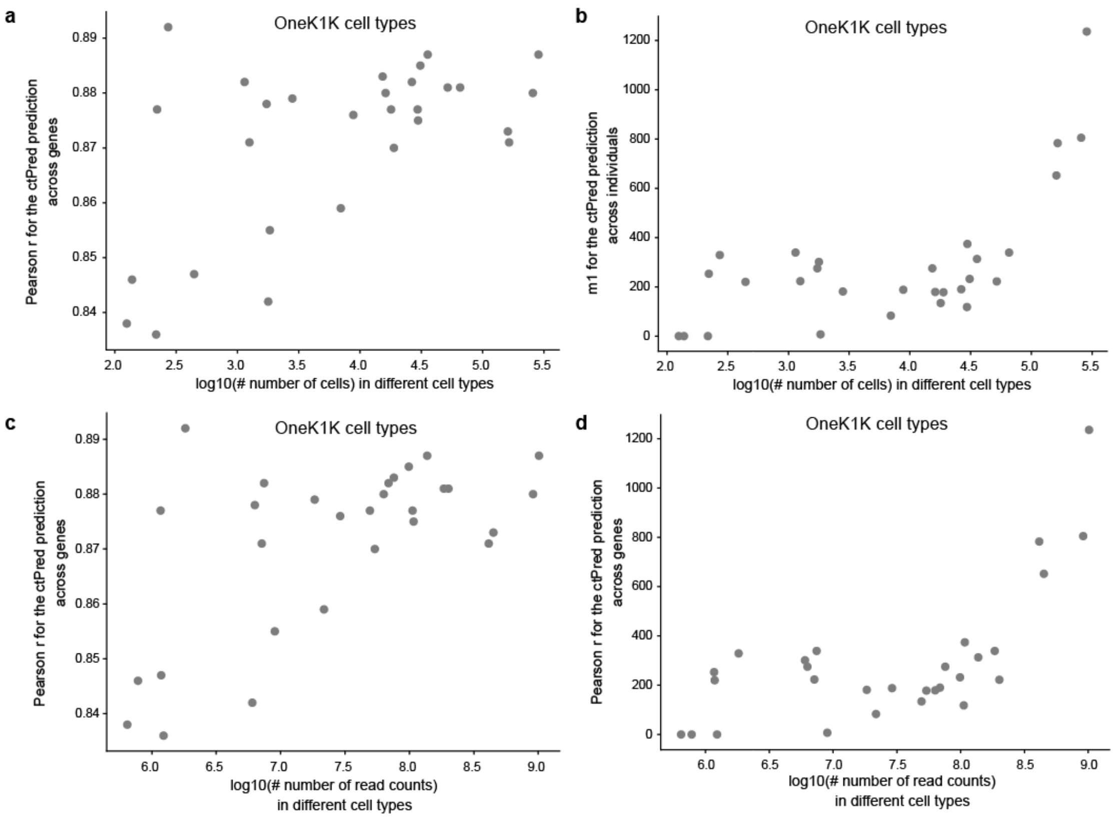

bioRxiv preprint doi: https://doi.org/10.1101/2024.11.11.623049; this version posted March 4, 2025. The copyright holder for this preprint (which was not certified by peer review) is the author/funder, who has granted bioRxiv a license to display the preprint in perpetuity. It is made available under aCC-BY 4.0 International license.

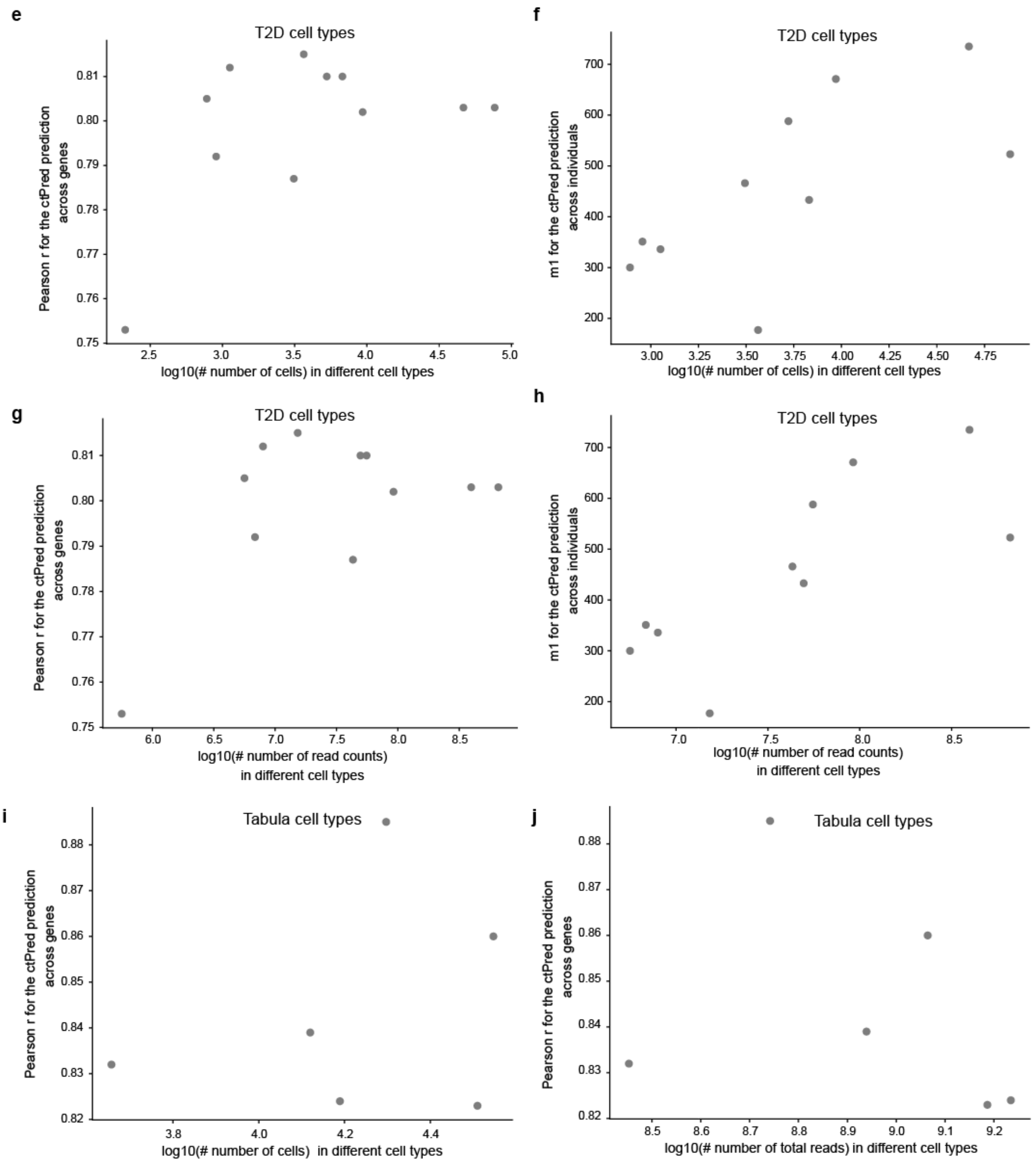

Supplementary fig. 7 a)-d) Prediction performance vs number of cells and read counts in OneK1K dataset. Scatter plot of ctPred prediction metrics (Pearson r for prediction across genes or m1 value for prediction across individuals) and cell numbers or total scRNAseq read counts in different cell types from OneK1K dataset. See supplementary table tab. 44. **e)-h) Prediction performance vs number of cells and read counts in T2D dataset.** Scatter plot of ctPred prediction metrics (Pearson r for prediction across genes or m1 value for prediction across individuals) and cell numbers or total scRNAseq read counts in different cell types from T2D dataset. See supplementary table tab. 44. **i)-j) Prediction performance vs number of**

bioRxiv preprint doi: https://doi.org/10.1101/2024.11.11.623049; this version posted March 4, 2025. The copyright holder for this preprint (which was not certified by peer review) is the author/funder, who has granted bioRxiv a license to display the preprint in perpetuity. It is made available under aCC-BY 4.0 International license.

cells and read counts in Tabula Sapiens dataset. Scatter plot of ctPred prediction metrics (Pearson r for prediction across genes for prediction across individuals) and cell numbers or total scRNAseq read counts in different cell types from Tabula Sapiens dataset. See supplementary table tab. 44.

Supplementary fig. 8: scRNA-seq pseudobulk data processing for ctPred training.

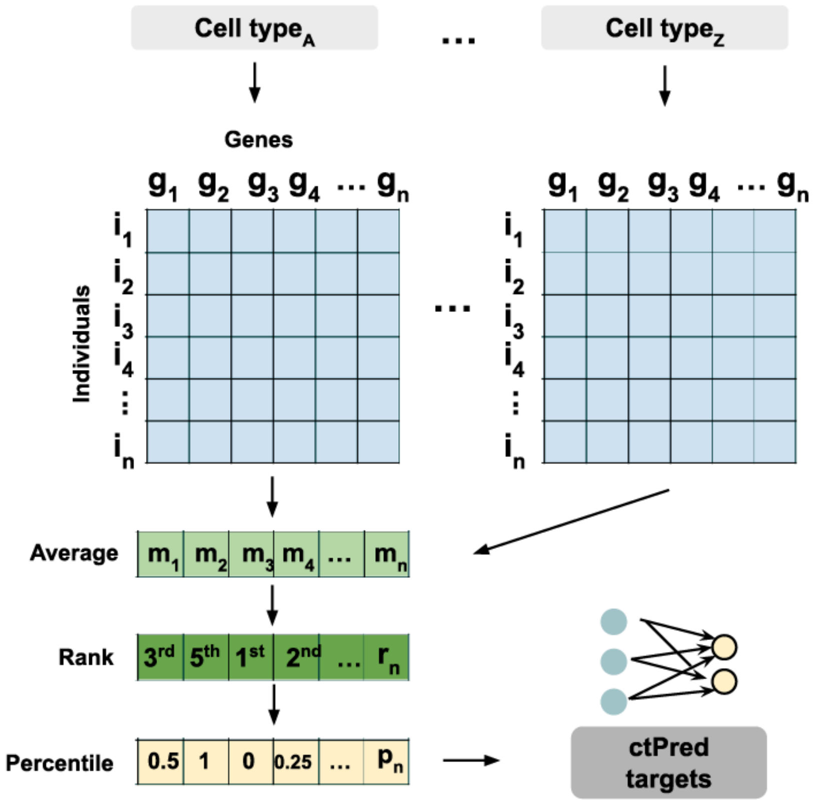

Supplementary fig. 8 The scRNA-seq processing into the target for ctPred model training.

Supplementary tables

Supplementary tables 1: General information (tables 1-3, tables 44-45). Table 1: cell types in T2D, OneK1K and Tabula Sapiens datasets. Table 2: number of genes trained and converged of PEN in the canonical TWAS framework. Table 3: ctPred prediction performance across genes in different cell types of three datasets. Table 44: ctPred prediction performance against the number of cells or total read counts per cell type for training in different cell types from T2D, OneK1K and Tabula Sapiens datasets. Table 45: Gene names of T2D silver-standard genes.

Supplementary tables 2: T2D TWAS results (tables 4-14). Table 4-14: T2D TWAS association z-score, effect sizes and p-values of tested genes in different cell types from T2D dataset.

bioRxiv preprint doi: https://doi.org/10.1101/2024.11.11.623049; this version posted March 4, 2025. The copyright holder for this preprint (which was not certified by peer review) is the author/funder, who has granted bioRxiv a license to display the preprint in perpetuity. It is made available under aCC-BY 4.0 International license.

Supplementary tables 3: SLE TWAS results (tables 15-43). Table 15-43: SLE TWAS association z-score, effect sizes and p-values of tested genes in different cell types from OneK1K dataset.
